# Orbital and Eyelid B-Cell Lymphoma: A Multicenter Retrospective Study

**DOI:** 10.3390/cancers12092538

**Published:** 2020-09-07

**Authors:** Gustavo Savino, Giulia Midena, Maria Antonietta Blasi, Remo Battendieri, Gabriela Grimaldi, Martina Maceroni, Fausto Tranfa, Pasquale Napolitano, Vittoria Lanni, Adriana Iuliano

**Affiliations:** 1 UOC Oncologia Oculare, Fondazione Policlinico Universitario A. Gemelli IRCCS, 00168 Roma, Italy; gustavo.savino@unicatt.it (G.S.); mariaantonietta.blasi@unicatt.it (M.A.B.); remo.battendieri@guest.policlinicogemelli.it (R.B.); martina.maceroni01@icatt.it (M.M.); 2 Istituto di Oftalmologia, Università Cattolica del Sacro Cuore, 00168 Roma, Italy; 3 Moorfields Eye Hospital NHS Foundation Trust, London EC1V 2PD, UK; gabriela.grimaldi@nhs.net; 4 Dipartimento di Neuroscienza, della Riproduzione e di Odontostomatologia, Centro di Patologia Orbitaria. Università “Federico II” di Napoli, 80138 Napoli, Italy; fausto.tranfa@unina.it (F.T.); p.napolitano3@studenti.unimol.it (P.N.); vittorialanni@hotmail.com (V.L.); adriana.iuliano@unina.it (A.I.)

**Keywords:** orbital neoplasms, ocular adnexal lymphoma, orbital lymphoma, eyelid lymphoma, ocular lymphoma prognosis, ocular lymphoma treatment, precision medicine

## Abstract

**Simple Summary:**

The treatment of orbital and eyelid B-cell lymphoma remains a field of progress. The aim of our study was to analyze patients diagnosed, staged and treated for orbital and eyelid B-cell lymphoma to assess clinical characteristics, treatment outcomes and recurrence patterns. We included in this study 141 cases of orbital and eyelid B-cell lymphoma. We found five lymphoma subtypes and we confirmed that the histopathologic subtype and the type of treatment were found to be the main factors influencing treatment outcome.

**Abstract:**

Background: The aim of this study was to analyze patients diagnosed, staged and treated for orbital and eyelid B-cell lymphoma (OEL). Methods: One hundred and forty-one cases of OEL were included in this study. Primary endpoints were to analyze the histopathologic findings, the main risk factors and the type of treatment and to correlate them with recurrence of OEL. The secondary endpoint was to determine the progression-free survival (PFS) time. Results: Extranodal marginal zone B-cell lymphoma was the most frequent subtype (66%), followed by small lymphocytic lymphoma (12.7%), diffuse large B-cell lymphoma (DLBCL) (9.2%), follicular lymphoma (6.6%), mantle cell lymphoma (4.3%) and Burkitt lymphoma (1.2%). The probability of relapse was influenced by the histopathologic subtype DLBCL (OR = 7.7, 95% CI 1.8–32.3) and treatment with chemotherapy (OR = 14.9, 95% CI 2.6–83.7). Multivariate analysis showed that the histopathologic subtype DLBCL and chemotherapy treatment retained statistical significance for a poorer PFS, with hazard ratios of 8.581 (*p* = 0.0112) and 9.239 (*p* = 0.0094), respectively. Conclusions: Five lymphoma subtypes were found in patients with OEL. The histopathologic subtype and the type of treatment were found to be the main factors influencing treatment outcome.

## 1. Introduction

Ocular adnexal lymphoma (OAL) refers to malignant lymphoproliferative diseases that may involve the orbit (orbital tissue and lacrimal gland), the eyelid and the conjunctiva.

Orbital lymphoma constitutes 46–74% of OAL and accounts for approximately 11% of all orbital masses [1,2]. Conjunctival and eyelid lymphomas represent 20–33% and 5–24% of OAL, respectively [3,4,5].

This study is focused on orbital and eyelid lymphoma (OEL). We have excluded patients affected by conjunctival lymphoma because of its peculiar distinction from orbital and eyelid lymphomas (OEL) in terms of biological behavior, clinical course and therapeutic regimen [6].

The majority of OELs are non-Hodgkin B-cell lymphomas and are observed more commonly in adults in the seventh decade of life. OEL accounts for 7% of all extranodal lymphomas and only 1% of all non-Hodgkin lymphomas. OELs are mainly unilateral, with bilateral OELs found in 7–24% of cases [2,3,4]. Most OELs are low-grade B-cell non-Hodgkin lymphomas, and approximately half are extranodal marginal zone B-celI lymphomas (EMZLs), previously also known as mucosa-associated lymphoid tissue (MALT) lymphomas [7,8]. Other common histopathologic subtypes of OEL are follicular lymphoma (FL), mantle cell lymphoma (MCL), diffuse large B-cell lymphoma (DLBCL) and small lymphocytic lymphoma (SLL) [4,7,8]. The majority (73%) of OELs arise as a primary orbital or eyelid disease, whereas 27% occur secondarily as a metastatic spread. Secondary OELs are generally assumed to be of similar histologic subtype as the systemic lymphoma; however, in a large retrospective study, patients initially diagnosed with low-grade orbital lymphoma were subsequently diagnosed with a dissimilar systemic high-grade lymphoma, due to a Richter transformation [9]. Few researchers have focused exclusively on the biology of OEL [10,11,12,13,14]. The main OEL genetic alterations for each histopathologic subtype mentioned above are reported in Table 1 [15,16,17,18,19,20,21,22,23,24,25,26,27,28,29,30].

Various studies have identified negative prognostic factors for OEL, including age greater than 60 years, lymph node involvement and elevated serum lactate dehydrogenase levels [28]. Several reports have also established the relationship between microorganism infection, mainly *Chlamydia psittaci*, and lymphoma [31,32,33].

Historically, all lymphomas, including OAL, were staged according to the Ann Arbor staging classification. Recently, the American Joint Committee on Cancer (AJCC) added a more specific and precise OAL (including OEL) TNM staging system in the eighth edition of its cancer staging manual [34].

The field of OEL is rapidly advancing because of progression in the understanding of tumor biology and pharmacology and the advent of targeted therapies. However, the treatment of OEL still remains a field of controversy. Currently, OELs are mostly treated using radiotherapy, immunotherapy, multi-agent chemotherapy or a combination of these treatment types [1,4]. Limited data have been reported in large cohorts of patients, homogenous in terms of staging and treatment. The aim of our study was to retrospectively analyze patients diagnosed, staged and treated for OEL in two Italian ocular oncology centers, to record risk factors (infection with *Helicobacter pylori*, *Chlamydia psittaci*, Hepatitis B Virus (HBV), Hepatitis C Virus (HCV) or history of rheumatoid arthritis, Sjógren’s syndrome or other connective tissue diseases) and to assess clinical characteristics, treatment outcomes and recurrence patterns.

## 2. Materials and Methods

### 2.1. Study Design

This study was a retrospective observational multicenter case series based on the data from two Italian ocular oncology centers: the Orbit Unit of the University “Federico II” of Naples, Naples, and the Orbit Unit of the “Fondazione Policlinico Gemelli IRCCS”, Rome.

The medical records of all patients with a histologic diagnosis of B-cell OEL involving the orbital adnexal region from 1 January 2008 through 31 December 2017 were identified and included in the study. We have excluded conjunctival lymphoma because the conjunctiva is a mucous membrane, which has its own lymphoid component, compared to the orbit and eyelids where lymphoid cells are physiologically absent. This conjunctival-associated lymphoid tissue protects the eye against foreign antigens and plays a direct role in the pathophysiology of conjunctival lymphoma. For this peculiar characteristic, for example, local immunotherapy with intralesional injections of interferon-α is the treatment of choice for conjunctival lymphomas, but it is not included in the standard treatments for OEL. All patients underwent diagnostic incisional biopsy, and all specimens were stained with hematoxylin and eosin and analyzed immunohistochemically for histopathologic examination. The local ocular oncology centers reviewed the samples and classified the specimens according to the World Health Organization Classification of Tumors of Haematopoietic and Lymphoid Tissues, Revised Fourth Edition. The study was carried out with approval from the Institutional Review Boards and the “Fondazione Policlinico Gemelli IRCCS” Ethics Committee (7202/18, ID:1942) and in adherence to the tenets of the Declaration of Helsinki. Patients were not directly involved in the design of this study.

### 2.2. Collected Data

The clinical collected data included age, gender, risk factors, symptoms, clinical findings, systemic involvement according to the eighth-edition AJCC TNM classification system, treatment modalities, response to therapy and survival duration.

The main risk factors analyzed were previous or current infection with *Helicobacter pylori*, *Chlamydia psittaci*, HBV and HCV; history of rheumatoid arthritis, Sjógren’s syndrome and connective tissue disease. Complete diagnostic workup of OEL included computed tomography (CT) and/or magnetic resonance imaging (MRI) of the orbital area, full-body positron emission tomography-computed tomography (PET-CT) and bone marrow biopsy. Only primary lymphomas were classified according to the AJCC TNM staging system. Complete ophthalmic examination included best corrected visual acuity, exophthalmometry, color vision testing, inspection and palpation of the eyelids and orbit, evaluation of ocular motility, intraocular pressure measurement and ophthalmoscopy.

### 2.3. Statistical Analysis

Primary endpoints analyzed were the correlation between histopathologic findings, the main risk factors, the type of treatment and recurrence of OEL. The secondary endpoint was to determine the progression-free survival (PFS) time. PFS was defined as the date of diagnosis to either the date of first relapse or progression after initial treatment, the date of death by any cause or the date of last contact, with the latter two being censored events.

The statistical analysis was carried out according to the usual methods of descriptive statistics: frequency distribution and percentages. Demographic and clinical data were described in terms of median. Associations between local recurrence and histopathologic findings, risk factors and treatment were evaluated using the chi-square test. Logistic regression analysis was used to identify whether the factors that were significant in the univariate analysis were still statistically significant in the multivariate analysis. In all cases, a *p* value of <0.05 was considered significant.

Survival analysis was carried out using the method described by Kaplan Maier. Univariate analysis using a log-rank test was performed with the following variables: risk factors, histopathology and treatment. Factors prognostic for PFS with a *p* value < 0.2 in the univariate analysis were studied in a multivariate analysis using the Cox proportional hazards model.

## 3. Results

### 3.1. Clinical Features

One hundred forty-one patients affected by B-cell OEL were included in the study. The main clinical and demographic characteristics for each histopathologic subtype are summarized in Table 2.

Without conspicuous differences among the different histopathologic subtypes, the main signs and symptoms reported were a mass in the orbit or eyelid, swelling, proptosis and globe displacement.

The majority of OELs were EMZLs, (66%, *n* = 93). In this group the median age was 65 years (±8.3), the disease was mainly unilateral (93.5%, *n* = 87) and primary (92%, *n* = 86) with a T2N0M0 staging in 80% of primary EMZLs (*n* = 69). Seventeen patients (18%) had risk factors, among whom eight patients had a diagnosis of HCV infection. Eighteen patients (12.8%) were diagnosed with small lymphocytic lymphoma (SLL) and the median age was 66 years (±9.2). The disease was unilateral in 13 cases, primary in almost all cases (89%, *n* = 16), and only a few patients (22%, *n* = 4) had risk factors. The majority of patients had a T2N3M0 (33%, *n* = 6) followed by a T2N0M0 (22%, *n* = 4) stage.

Thirteen patients (9.2%) were diagnosed with DLBCL. The median age of the group was 68 years (±5.6), with higher male prevalence (62%, *n* = 8). The disease was unilateral in all cases, primary in eight cases and staged as T2N0M0 in 62% of cases (*n* = 8); secondary DLBCL was diagnosed in five patients (38%). More than 50% of patients showed risk factors (*n* = 7), and the main risk factor was infection by HCV (46%).

Nine patients (6.6%) had a diagnosis of FL. The median age of the group was 66 years (±9.1). The disease was unilateral and primary in all cases; T2N1bM0 was the most common staging level (45%, *n* = 4), and HCV infection was recorded in five patients (56%).

Six patients were diagnosed with MCL. The median age of the group was 71 years (±3.1), with higher male prevalence (83%, *n* = 5). The disease was unilateral in four cases and bilateral in two cases. The majority of these patients had a primary disease (83%, *n* = 5) with T2N0M0 stage, and no one showed risk factors. Just two cases were diagnosed as Burkitt lymphoma (BL). Both patients were in their forties, and the disease was secondary and unilateral in both cases.

### 3.2. Treatment

Of the 86 primary EMZLs, 63 (73%) were treated with external beam radiation therapy (EBRT), 13 (15%) with chemotherapy using the CHOP (Cyclophosphamide, Hydroxydaunorubicin, Vincristine and Prednisone) regimen or unspecified chemotherapy and 5 (5.8%) with Rituximab (MabThera, Roche, Basel, Switzerland). A combination regimen of chemotherapy and immunotherapy (CHOP and Rituximab) was used only in two cases (2.2%) of primary EMZL. In three cases (4%), patients refused any intervention and chose to undergo regular controls. The seven patients with a diagnosis of secondary EMZL were all treated with chemotherapy, which was combined with EBRT in three cases. Among the 16 patients with primary SLL, 1 received EBRT, 5 underwent chemotherapy and a combination regimen was used in the remaining 10 patients (CHOP and EBRT for 9 patients; Rituximab and EBRT for 1 patient). The two cases with secondary SLL were both treated with chemotherapy and EBRT. Among the eight primary DLBCLs, three (37.5%) were treated with EBRT, one (12.5%) with chemotherapy, one (12.5%) with immunotherapy and three (37.5%) with a combination regimen (CHOP and EBRT for two patients; Rituximab and EBRT for one patient). The five secondary DLBCLs were all treated with chemotherapy, associated with EBRT in three cases and with EBRT and immunotherapy in two cases. The nine FL cases were treated with EBRT in two cases (22%), with chemotherapy in five cases (56%) and with immunotherapy in two cases (22%).

All patients with primary MCL underwent EBRT (83%, *n* = 5), and the only case with secondary MCL was treated with EBRT in combination with chemotherapy. The two cases of BL were treated with chemotherapy.

### 3.3. Treatment Outcome and Survival

**Figure 1:**
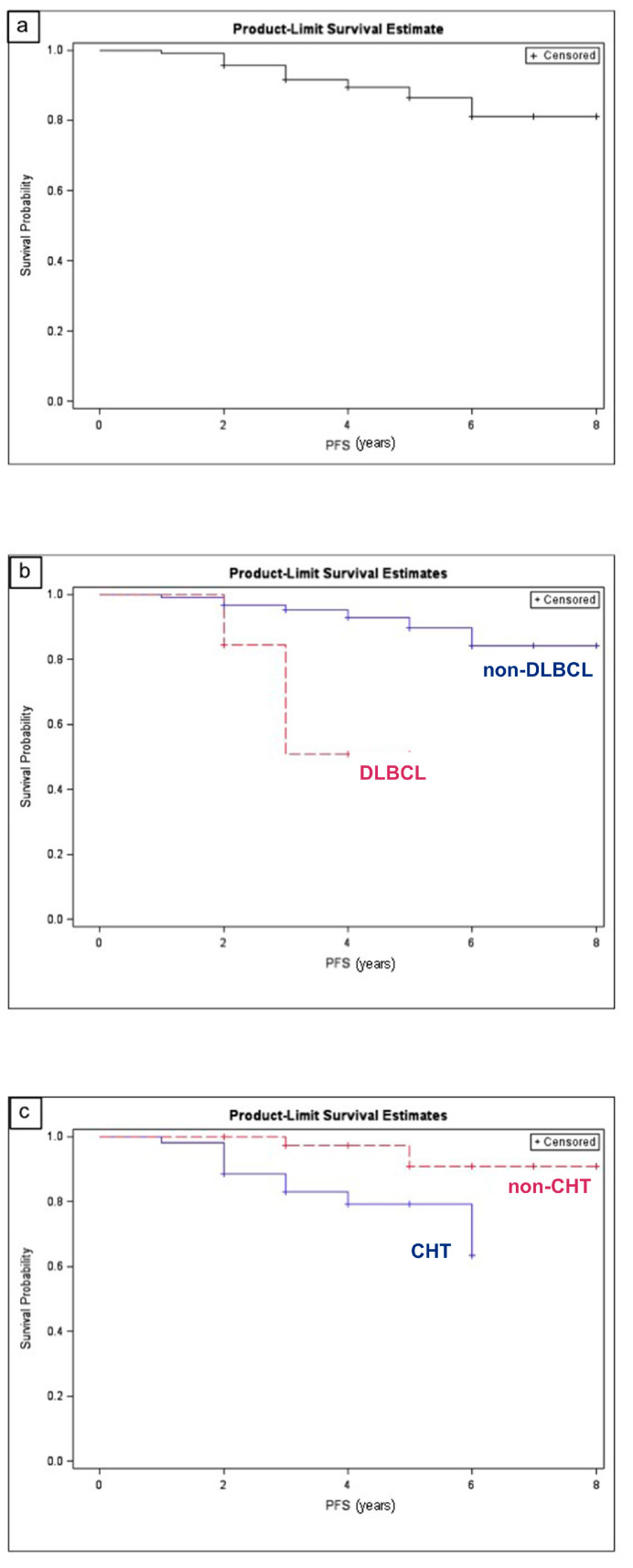
Kaplan–Meier estimate of progression-free survival for patients with orbital and eyelid lymphoma (OEL). (**a**) Kaplan–Meier estimate of progression-free survival of the whole sample. (**b**) Kaplan-Meier estimate of progression-free survival of patients with diffuse large B-cell lymphoma (DLBCL) vs. patients with other histological subtypes of OEL. (**c**) Kaplan–Meier estimate of progression-free survival of patients treated with chemotherapy vs. patients treated without chemotherapy. (Graphic program: SAS V.9.3, SAS Institute Inc., Cary, NC, USA).

The median follow-up was 48 months (±7.3). Relapse was observed in 12 patients (8.5%). Among them, five patients (41.6%) had a diagnosis of EMZL, with the lymphoma being secondary in one case, in a metastatic stage in three cases (with lung involvement in two patients and spleen metastases in one) and localized in one case; this last patient was treated with Rituximab and recurred in five years. Four relapsed cases (33.4%) belonged to the DLBCL subtype; in two cases the disease was localized, and patients were treated with monotherapy; in the other two cases the lymphoma was secondary, in a systemic widespread disease. Three patients (25%) had a diagnosis of SLL; the disease was widespread and treated with combined therapy (chemotherapy and EBRT) in all cases. The probability of relapse seemed to be related to the histopathologic subtype DLBCL (odds ratio = 7.7, 95% CI 1.8–32.3) and to chemotherapy treatment (odds ratio = 14.9, 95% CI 2.6–83.7). The median PFS was 3 ± 1.4 years (Figure 1a).

Table 3 shows the PFS along with univariate and multivariate analyses. On multivariate analysis, the histopathologic subtype DLBCL (Figure 1b) and chemotherapy treatment (Figure 1c) retained statistical significance for a poorer PFS, with hazard ratios of 8.581 (*p* = 0.0112) and 9.239 (*p* = 0.0094), respectively.

## 4. Discussion

This multicenter study retrospectively analyzed patients with a diagnosis of OEL, referred to two Orbit Units homogenous with regard to diagnostic and histopathologic criteria, staging system and therapeutic approaches. Patients affected by conjunctival lymphoma were excluded because of its peculiar distinction from OEL in terms of biological behavior, clinical course and therapeutic regimen.

EMZL, as previously reported, was the most frequent subtype: 66% of our population (93 cases), followed by SLL at 13% (18 cases), DLBCL at 9.2% (13 cases) and FL at 6.4% (9 cases). Low-incidence lymphomas were MCL at 4.3% (six cases) and Burkitt lymphoma at 1.1% (two cases). Other authors reported similar subtype rates [4]. In other geographic areas, the incidence of EMZL was higher and the subtype distribution different [4]. We found that most of our patients were elderly people (median age: 63 years). Moreover, patients with MCL tended to be slightly older than patients with EMZL, FL and DLBCL, confirming previous reports [1,2,4,23]. The old age of patients may indeed play a role in the pathogenesis of OEL. Recent studies have underlined how immunosenescence plays an essential, but poorly defined, role in the development of lymphomas [35]. Furthermore, immunosenescence is associated with a complex dysfunction that increases sensitivity to infections, and some reports have suggested a relationship between age-related immune dysregulation, OEL subtypes and infections [35,36]. In our study, we did not find any statistically significant correlation between presumed risk factors (infection with *Helicobacter pylori*, *Chlamydia psittaci*, HBV, HCV or history of rheumatoid arthritis, Sjógren’s syndrome or other connective tissue diseases) and recurrence rate, but we recorded a higher incidence of HCV infection in FLs (55.5%) and DLBCLs (38.5%), particularly in those cases with a more diffuse dissemination at the onset (80%). These data seem to support Strianese et al. who hypothesized that the long-term antigenic stimulation provided by HCV infection may elicit host immune responses able to promote and sustain clonal B-cell expansion [37].

Regarding the therapeutic regimen, in our study 73% of patients with EMZL received EBRT. In most cases, the disease was in a T2N0M0 stage and no relapse was noted. This finding is consistent with the current literature that supports the use of EBRT for most cases of primary OEL, especially for low-grade lymphomas such as EMZL, which represented the majority of cases in our series [38,39,40,41,42]. A newly published review by the American Academy of Ophthalmology on treatment of OEL has documented that EBRT has a very good effect on local control, disease-free survival and overall survival in patients with EMZL [43]. EBRT is also the treatment of choice for MCL, which, despite not being a low-grade lymphoma, has been found in some studies to be particularly radiosensitive [23,41,44,45]. Cases of MCL (83%) were treated with EBRT and showed a good response without any relapse.

As for chemotherapy, it is generally acknowledged that it is usually indicated for more aggressive OEL histologic subtypes with potential for future systemic involvement or with existing disseminated disease [24]. Complete response rates of 60% to 80% and predicted five-year survivals greater than 55% have been obtained with chemotherapy or combined therapy regimens [24]. We found that chemotherapy or combined therapy regimens were the treatment modalities of choice for FL and DLBCL.

In particular, all FL patients were found to have lymph node involvement at the time of diagnosis; hence, chemotherapy was the treatment of choice. It should be noted Rasmussen et al. reported that EBRT provided excellent disease control in primary ocular adnexal FL [11]. Nevertheless, in our series chemotherapy was preferred due to the fact that disseminated disease usually exhibits frequent relapses [21,46]. Interestingly, no patient with FL relapsed in our series, following chemotherapy treatment.

DLBCL received combined therapy regimens in the 61.5% of cases, as previously reported [47]. In our series, 31% of patients with DLBCL showed recurrence, and we found a statistically significant correlation (odds ratio = 7.7, 95% CI 1.8–32.3) between recurrence rate and the DLBCL histologic subtype. Moreover, this histologic subtype retained statistical significance for a poorer PFS, with a hazard ratio of 8.581 (*p* = 0.0112). DLBCL is known to be a heterogeneous entity with considerable variability in clinical features, morphology and genetics [48]. As a consequence, the response to chemotherapy is variable and difficult to predict. Several studies have, without success, attempted to elucidate whether these features, either clinical, morphologic or genetic, may improve prognostication [49,50]. Not surprisingly, the overall probability of relapse seemed to be related to chemotherapy treatment (odds ratio = 14.9, 95% CI 2.6–83.7) which, in turn, retained statistical significance for a poorer PFS, with a hazard ratio of 9.239 (*p* = 0.0094). This finding confirms the fact that chemotherapy was indeed indicated and utilized for more aggressive and disseminated tumors.

Immunotherapy with Rituximab, as sole therapy, was used only in selected cases, namely very old patients with primary low-grade OEL in whom orbital involvement was not causing any quality-of-life issues [43,51]. These patients did not have HBV infection, and the maintenance treatment with Rituximab was done easily with very little morbidity; recurrence was recorded only in one case, after five years. On the other hand, Rituximab in combination with chemotherapy and/or EBRT showed good results in the treatment of high-grade OEL, without any recurrence. Moreover, we reserved Rituximab for those patients whose disease relapsed after initial EBRT, but these data were not included in the present work.

Regarding the TNM staging system, the AJCC staging classification allowed a precise characterization of the extent of local disease, and no additional prognostic factors were required for stage grouping [52,53]. No association was detected in our study between the T category and recurrence. The AJCC proposed additional factors recommended just for clinical care: the International Prognostic Index (IPI), tumor cell growth fraction (Ki-67, MIB-1) and lactate dehydrogenase level [34,35,36,37,38,39,40,41,42,43,44,45,46,47,48,49,50,51,52,53,54,55]. Nonetheless, these factors were not considered in this series as complete data were available only for a small percentage of studied patients.

## 5. Conclusions

Our study suggested that the most frequent subtypes of OEL, in terms of prevalence, are EMZL, SLL, DLBCL, FL, MCL and BL. The histopathologic subtype and the type of treatment were found to be the main predictors for treatment outcome, as the DLBCL histopathologic subtype and chemotherapy correlated with a higher risk of recurrence and with a poorer PFS. Further prospective studies are warranted to better define the role of all prognostic tools defined by the AJCC and to move toward a tailored therapeutic approach so as to apply the most effective treatment modality in the individual patient, especially in consideration of such a heterogeneous disease as OEL.

## Figures and Tables

**Table 1:** Genetic characteristics of the most common OEL subtypes.

Lymphoma Subtypes	Genetic Alterations
EMZL	- t(11;18)(q21;q21)in 15–40% [15] - t(14;18)(q32;q21) in 24% [15] - t(3;14)(p14.1;q32) in 20% [16,17,18] - Trisomy 3, 18 [16,17,18]
FL	- t(14;18)(q32;q21) in 76%, resulting in the expression of BCL-2 [19,20] - p53 gene mutations and c-*myc* rearrangement in high-grade cases [20,21]
MCL	- t(11;14)(q13;q32) in almost all cases, resulting in cyclin D1 overexpression [22,23,24] - p53 gene mutations and c-*myc* rearrangement in high-grade cases [22,23,24]
DLBCL	- Bcl-6 gene rearrangements in 40% [24] - Bcl-2 gene rearrangements in 25% [24] - C-*myc* gene rearrangements extremely rare [25,26]
SLL	- del(13q) in 55% [28,29] - Trisomy 12 [28,29]

EMZL: extranodal marginal zone lymphoma; FL: follicular lymphoma; SLL: small lymphocytic lymphoma; MCL: mantle cell lymphoma; DLBCL: diffuse large B-cell lymphoma.

**Table 2:** Clinical features and histopathologic subtypes of patients with OEL.

Clinical Features and Histopathologic Subtypes	EMZL	SLL	DLBCL	FL	MCL	BL
**No. of patients**	93	18	13	9	6	2
**Gender (Male:Female)**	47:46	10:8	8:5	4:5	5:1	1:1
**Median age at presentation (SD)**	65 (8.3)	66 (9.2)	68 (5.6)	66 (9.1)	71 (3.1)	40 (0.5)
**Laterality (Unilateral:Bilateral)**	87:6	13:5	13:0	18:0	4:2	2:0
**Location:**						
**- Orbital tissue**	76	18	11	9	4	2
**- Lacrimal gland**	14	-	2	-	2	-
**- Eyelid**	3	-	-	-	-	-
**Disease presentation:**						
**- Primary OEL**	86	16	8	9	5	-
**- Secondary OEL**	7	2	5	-	-	2
**No. of patients with risk factors:**	17	4	7	5	-	-
**Symptoms:**						
**- Mass**	80	7	11	6	6	2
**- Swelling**	72	13	13	7	4	2
**- Proptosis**	31	2	11	2	1	-
**- Diplopia**	10	1	9	1	-	2
**- Ptosis**	14	-	1	-	-	-
**Signs:**						
**- Proptosis**	47	9	11	3	4	2
**- Globe displacement**	63	11	10	1	1	2
**- Limited motility**	27	5	9	5	2	2
**- Ptosis**	34	-	-	-	-	-
**- Epiphora**	20	2	2	1	1	-

EMZL: extranodal marginal zone lymphoma; FL: follicular lymphoma; SLL: small lymphocitic lymphoma; MCL: mantle cell lymphoma; DLBCL: diffuse large B-cell lymphoma; BL: Burkitt lymphoma. SD: standard deviation.

**Table 3:** Univariate and multivariate analyses for progression-free survival (PFS).

Category	Variable	Univariate		Multivariate	
		Median Survival Time (SD)	*p*-Value	HR (95%)	*p*-Value
**Histopathology**	EMZL	60 (2)	0.1973	2.853	0.2014
	SLL	66 (3)	0.5244	-	-
	DLBCL	36 (0.5)	**0.0004**	**8.581**	**0.0112**
	FL	51 (2.5)	0.2601	-	-
	MCL	46 (2.6)	0.4530	-	-
	BL	36 (0.7)	0.6703	-	-
**Risk factors**	HCV	36 (1.1)	0.3231	-	-
	HBV	43 (2.1)	0.5720	-	-
	Others	43 (3.1)	0.3435	-	-
**Treatment**	EBRT	68 (3.3)	0.3531	-	-
	CHT	63 (3.2)	**0.0025**	**9.239**	**0.0094**
	Immunotherapy	60 (2.2)	0.8556	-	-

EMZL: extranodal marginal zone lymphoma; SLL: small lymphocytic lymphoma; DLBCL: diffuse large B-cell lymphoma; FL: follicular lymphoma; MCL: mantle cell lymphoma; BL: Burkitt lymphoma; EBRT: external beam radiation therapy; CHT: chemotherapy. HR: hazard ratio; SD: standard deviation.
